# Carbon dioxide fluxes in a farmland ecosystem of the southern Chinese Loess Plateau measured using a chamber-based method

**DOI:** 10.7717/peerj.8994

**Published:** 2020-04-27

**Authors:** Fengru Fang, Xiaoyang Han, Wenzhao Liu, Ming Tang

**Affiliations:** 1 College of Forestry, State Key Laboratory of Soil Erosion and Dryland Farming on the Loess Plateau, Institute of Soil and Water Conservation, Northwest A&F University, Yangling, China; 2 State Key Laboratory of Conservation and Utilization of Subtropical Agro-bioresources, Lingnan Guangdong Laboratory of Modern Agriculture, Guangdong Key Laboratory for Innovative Development and Utilization of Forest Plant Germplasm, College of Forestry and Landscape Architecture, South China Agricultural University, Guangzhou, China

**Keywords:** Net ecosystem exchange, Soil respiration, Temporal variation, Temperature sensitivity, Loess Plateau

## Abstract

**Background:**

Farmland accounts for a relatively large fraction of the world’s vegetation cover, and the quantification of carbon fluxes over farmland is critical for understanding regional carbon budgets. The carbon cycle of farmland ecosystems has become a focus of global research in the field of carbon dynamics and cycling. The objectives of this study are to monitor the temporal variation in the net ecosystem exchange (NEE) and soil respiration in a spring maize (*Zea mays* L.) farmland ecosystem of the southern Loess Plateau of China.

**Methods:**

A fully automated temperature-controlled flux chamber system was adopted in this study. The system contained nine chambers for CO_2_ flux measurements, and three treatments were conducted: with and without maize plants in the chamber, as well as a bare field. Observations were conducted from June to September 2011. This time period covers the seedling, jointing, heading, grain filling, and ripening stages of spring maize. Other factors, such as air temperature (Ta), soil temperature (Ts), soil water content (SWC), photosynthetically active radiation (PAR), and precipitation (P), were simultaneously monitored.

**Results:**

There was observed diurnal variation in the NEE of the maize ecosystem (NEE-maize). A short “noon break” occurred when the PAR intensity was at its maximum, while soil respiration rates had curves with a single peak. During the overall maize growth season, the total NEE-maize was –68.61 g C m^−2^, and the soil respiration from the maize field (SR-maize) and bare field (SR-bare field) were 245.69 g C m^−2^ and 114.08 g C m^−2^, respectively. The temperature sensitivity of soil respiration in the maize field exceeded that in the bare field. Significant negative correlations were found between the NEE, PAR, and temperature (all *p*-values < 0.01), with both Ta and PAR being the primary factors that affected the CO_2_ fluxes, collectively contributing 61.7%, 37.2%, and 56.8% to the NEE-maize, SR-maize, and SR-bare field, respectively. It was therefore concluded that both meteorological factors and farming practices have an important impact on the carbon balance process in corn farmland ecosystems. However, it is necessary to conduct long-term observational studies, in order to get a better understanding of the driving mechanism.

## Introduction

The increase in atmospheric carbon dioxide (CO_2_) concentration caused by global climate change has elicited universal concern, especially with respect to the CO_2_ exchange flux between terrestrial ecosystems and the atmosphere. Terrestrial ecosystems are important in the global carbon cycle, and ecosystem respiration and gross photosynthesis are the major pathways of carbon exchange between these ecosystems and the atmosphere (Baldocchi et al., 2001; Yu et al., 2006; Wagle et al., 2017). The net ecosystem exchange (NEE) is the result of gross primary productivity (GPP) in combination with ecosystem respiration (R_eco_), and positive and negative values represent CO_2_ assimilation and release, respectively (Wang et al., 2013a). Croplands are strong contributors to the terrestrial carbon budget, and agricultural soil has been considered one of the largest emitters of greenhouse gases (Huang et al., 2007; Smith et al., 2010). Moreover, croplands are largely influenced by human activities (e.g., irrigation, farming, and fertilization), and the CO_2_ exchange flux between cropland ecosystems and the atmosphere is likely affected by various factors (Li et al., 2006; Lei & Yang, 2010). Thus, the carbon cycle of farmland ecosystems is an important issue in the rapidly advancing field of global carbon cycle research (Hutchinson, Campbell & Desjardins, 2007). Hence, accurate measurements of CO_2_ fluxes are particularly significant for China because the country contains the third largest area of farmlands following forests and grasslands (Liu et al., 2005).

Several methods have been developed for observing and determining the carbon flux in terrestrial ecosystems (Yu, Wang & Zhu, 2011). The micro-meteorological and chamber methods are the most widely used, while the eddy covariance method is considered as the standard micro-meteorological method for measuring ecosystem carbon and water fluxes (Baldocchi, 2003). However, the basic assumptions of the eddy covariance method, which include an underlying surface of a certain scale (flat and uniform) and equilibrium hydrothermal conditions, are not always satisfied. Hence, there are inevitable uncertainties in flux observations. The chamber method is widely used for the direct measurement of CO_2_ flux in smaller-scale ecosystems, especially for grasslands and croplands (Davidson et al., 2002; Balogh et al., 2007). Many studies have compared the chamber method with eddy covariance and reported similar or higher CO_2_ fluxes than found in chamber-based observations (Liang et al., 2004; Ohkubo et al., 2007; Nagy et al., 2011; Riederer, Serafimovich & Foken, 2014). Myklebust, Hipps & Ryel (2008) recommended the use of the eddy covariance, chamber, and gradient methods for measuring soil CO_2_ flux. Wang et al. (2013a) also indicated that an automatic chamber is a suitable alternative to the eddy covariance technique, and they reported good agreement between the NEE fluxes measured in cotton and wheat fields using both techniques. Morton & Heinemeyer (2018) recently assessed and highlighted the importance of including shrub vegetation in chamber flux calculations. Therefore, the CO_2_ flux measured by the chamber method can be used to obtain a relatively accurate flux value by using a suitable coefficient that can expand the application range of eddy correlation method observations (Lucas-Moffat et al., 2018). This offers an important complement for large-scale carbon exchange research (Hoffmann et al., 2015).

The Loess Tableland, located in the central and southern regions of the Loess Plateau, is an important crop production base in northwest China. Spring maize (*Zea mays* L.) and winter wheat are two traditional food crops cultivated there that have considerable acreages. They play a vital role in ensuring regional food security and maintaining the carbon balance (Liu et al., 2010). The carbon dynamics of a maize cropland have typically been measured using the eddy covariance technique at the ecosystem scale (Jans et al., 2010; Du & Liu, 2013; Vote, Hall & Charlton, 2015; Gao et al., 2017; Poyda et al., 2019), but few systematic studies of CO_2_ flux of the small rainfed spring maize fields of the Loess Plateau have been conducted using the chamber method. Zhang et al. (2011) used the dynamic chamber system to determine soil respiration only in wheat ecosystems, and they did not consider the absorption of CO_2_ due to plant photosynthesis. Continuous and long-term measurements are still necessary to explore the temporal variations in carbon flux components in maize fields and how they are controlled by climatic variables (Lei & Yang, 2010).

The objectives of this study are as follows: (1) to investigate the temporal variation of NEE and soil respiration (SR) rates in a maize farmland and bare field during the growing season; (2) to compare the carbon exchange capacity among different growth stages of spring maize plants; and (3) to explore the factors that affect carbon flux and their mechanisms in maize farmland ecosystems. Given the pressing need to better understand the characteristics of CO_2_ flux and its influencing factors on typical land use patterns, this work provides insight into the study of regional water and carbon processes, as well as carbon source/sink relationships.

## Materials and Methods

### Study site

This study was conducted at the Changwu Agro-Ecological Experiment Station on the Chinese Loess Plateau (35°12′N, 107°40′E, 1,200 m a.s.l.). Regional climate is driven by the semi-arid continental monsoon. The annual mean temperature is 9.1 °C, and the mean annual precipitation is 584 mm, with an annual mean potential evapotranspiration of 949 mm (1957–2012; Han, Liu & Lin, 2015). The Loess Tableland is a typical rain-fed agricultural area; hence, precipitation is the sole water resource for crop growth. The soils are *Cumuli-Ustic Isohumosols*, according to the Unified Soil Classification System (Gong, Zhang & Chen, 2007), a type that is porous and has a high-water holding capacity. However, this soil type suffers from moisture deficits under uneven rainfall and high evaporation conditions. The soil pH value is approximately 8.4, with a soil organic matter content of approximately 3% (Han et al., 2016).

### Experimental design

There are three types of fluxes in the field under observation: (1) NEE of carbon in the maize field (NEE-maize, with maize plants in the chamber); (2) soil respiration in the maize field (SR-maize, no plants in the flux box and covering of only the inter-row soil); and (3) soil respiration in the bare field (SR-bare field, with no tillage and regular weed removal). CO_2_ flux is the general term used for the NEE and SR of the maize ecosystem. The soil organic carbon (SOC) of 0–20 cm depth layers in the maize and bare fields were not significantly different, and the average values were 6.85 ± 0.61 gkg^−^^1^ and 6.34 ± 0.68 gkg^−^^1^, respectively (*n* = 6).

The spring maize variety used in the experiment was Xianyu 335, and its sowing time was April 28, 2011. The growth stages of corn primarily include the seedling (May 7 to June 10), jointing (June 11 to June 30), heading (July 1 to July 15), grain filling (July 16 to July 31), and ripening (August 1 to September 15) periods. The growth stages were classified according to the climatic and maize growth characteristics of this region (Liu et al., 2010). Fertilization is conducted every year during the sowing period. The fertilization standard applied was 300 kg ha^−^^1^ of nitrogen and 750 kg ha^−^^1^ of phosphate fertilizer.

### Field measurements of CO_2_ fluxes

**Figure 1:**
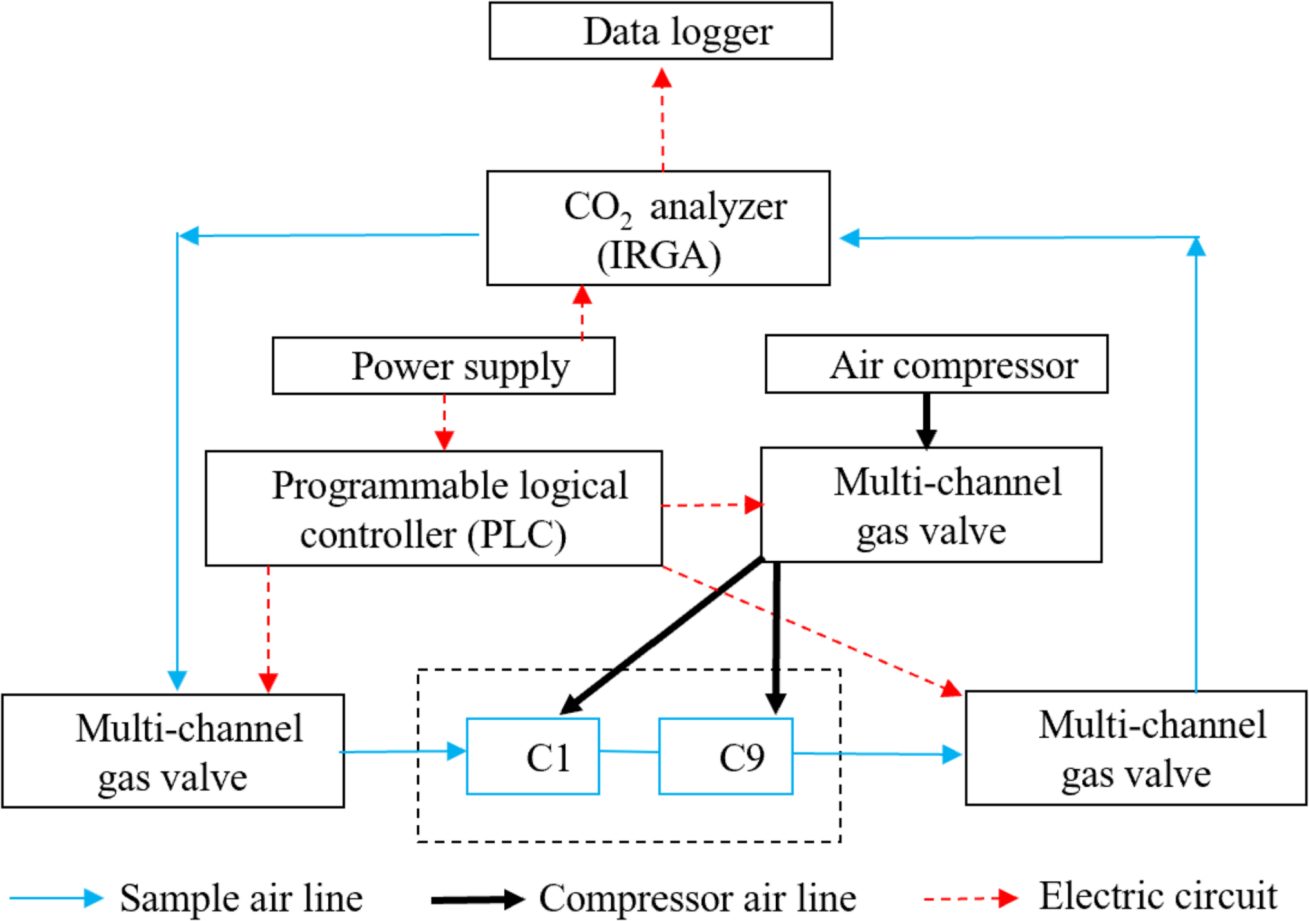
The automated multi-chamber system.

The CO_2_ fluxes were continuously measured in the maize field and bare field plots (10 m ×10 m) at intervals of one hour using a multi-channel automated chamber system (Fig. 1). There were three chambers per treatment, as described in our previous study (Han et al., 2016). The chambers (50 ×50 × 50 cm, L × W × H) were made of transparent plexiglass, with lids hinged at the sidewalls so they could be opened. In each chamber, there was a telescopic cylinder driven by high pressure from a compressor that opened and closed the lid, with a small fan used to mix the air when the lid was closed (Liang, Inoue & Fujinuma, 2003; Han et al., 2016). The air samples were pumped from one closed chamber into an infrared gas analyzer (IRGA, Li-820, Li-Cor, Lincoln, USA) to measure their CO_2_ concentration through a multi-channel valve connected with a desiccant tube, a filter, and a flow controller. A programmable logical controller (PLC, Master-K120S, LG, Korea) was used to control the solenoid valves, to open and close the target chambers, and to circulate the gas samples (Zhang et al., 2011; Han et al., 2016). Each chamber was closed for three minutes for measurements, and the flow rate was controlled at 1 L min^−^^1^ (Drewitt et al., 2002). After nine chambers were closed in sequence, the next measurement cycle was performed. The CO_2_concentration was measured continuously using an infrared gas analyzer (IRGA) and recorded using a data logger at ten second intervals (CR1000, Campbell Scientific, Logan, USA).

The automated chamber system has been tested repeatedly and has been proven to work well under field conditions with high precision and stability (Steduto et al., 2002; Liang, Inoue & Fujinuma, 2003; Zhang et al., 2011; Zhou et al., 2012). First, the chambers were made of transparent plexiglass with approximately 95% transmission, which allows plants in the chamber to perform photosynthesis normally, thus ensuring the accuracy of the NEE measurements. Second, the chambers were operated automatically. This provides the ability to collect averaged data of continuous observations during both the daytime and nighttime, as well as measurements during extreme weather, such as rainfall. Third, the collected gas was directly transferred from the infrared gas analyzer without the need for subsampling, which is more ideal than gas chromatography methods. Moreover, the small fan in the chamber was able to adjust the temperature and mix the gas inside the chamber during measurements, thus reducing the effect of temperature on CO_2_ flux.

Soil temperatures (Ts) at depths of 10- and 20-cm were measured using a thermocouple thermometer. The soil water content (SWC) at a depth of 10 cm was measured using a Hydra Probe (SDI-12, Stevens, USA). Other variables, such as air temperature (Ta), photosynthetically active radiation (PAR), and precipitation (P), were simultaneously monitored and recorded at the Changwu Agro-Ecological Experiment Station located approximately ten meters from the field site.

### Data processing

#### Calculation of CO_2_ flux

CO_2_ fluxes (NEE and SR) were calculated using Eq. (1) (Davidson, Belk & Boone, 1998; Steduto et al., 2002; Zhang et al., 2011; Han et al., 2016):

(1) \begin{eqnarray*}A= \frac{dc}{dt} \frac{V}{S} \frac{P}{R{T}_{a}} \end{eqnarray*}

where *A* is the CO_2_ flux in a certain area during a period of time, (μmol m^−^^2^ s^−^^1^); *dc/dt* is the change rate in CO_2_ concentrations; *V* is the volume of the chamber (m^3^); *S* is the ground surface area enclosed by the chamber (m^2^); *P* is the atmospheric pressure inside the chamber (kPa); *R* is a universal gas constant (8.3144 ×10^−^^3^ kPa m^3^ mol^−^^1^ K^−^^1^), and *T*_*a*_ is the air temperature inside the chamber (K). Diurnal and seasonal variations of the NEE and soil respiration were analyzed using two different datasets. Specifically, the diurnal variation was analyzed based on the hourly data, while the seasonal variation was based on the daily data.

#### Calculation of the Q_10_

An exponential curve was used to investigate the relationship between soil respiration and temperature as follows (Phillips et al., 2011):

(2) \begin{eqnarray*}SR=\mathrm{\alpha }{ e}^{ \mathrm{\beta }{T}_{s}}\end{eqnarray*}

where SR is the soil respiration rate (mol CO_2_ m^−^^2^ s^−^^1^), which is A in Eq. (1); *T*_*s*_ is the soil temperature (°C); and *α* and *β* are estimated parameters when SR is a known quantity.

A Q_10_ value was used to represent the corresponding multiples of soil respiration increase when the soil temperature was increased by 10 °C intervals using the form of Phillips et al. (2011):

(3) \begin{eqnarray*}{Q}_{10}={ e}^{10\mathrm{\beta }}\end{eqnarray*}

where *β* is the parameter from Eq. (2). Thus, Q_10_ could be used as a sign of temperature sensitivity for soil respiration.

#### Carbon in grains

It was hypothesized that all of the maize straws were returned to the field, and the carbon in the grains (C_*gr*_, gC m^−^^2^) was estimated using the crop yield (Y) as follows (Li et al., 2006; Lei & Yang, 2010; Wang et al., 2013b):

(4) \begin{eqnarray*}{C}_{gr}=(1-{W}_{gr}) {f}_{c}Y\end{eqnarray*}

where *Wgr* is the grain water content (0.155 for maize); *f*_*c*_ is the fraction of carbon in the grain (0.447 for maize); and *Y* is the grain yield (Li et al., 2006; Lei & Yang, 2010).

### Statistical analysis

Multiple comparisons and a one-way analysis of variance (ANOVA) was performed to evaluate the significance level of the differences in the CO_2_ fluxes among the treatments and different growth stages. The Pearson correlation coefficients were calculated to determine the significance of the correlations between CO_2_ fluxes and the influencing variables (Ta, Ts, PAR, P, and SWC). These statistical analyses were conducted using SPSS19.0 software (IBM SPSS Statistics, Chicago, USA). A principal component analysis (PCA) was conducted using CANOCO 5.0 to quantify the relative contribution of the influencing factors to the changes in the soil CO_2_ fluxes. All figures were drawn using SigmaPlot 12.5 software (Systat Software, San Jose, USA).

## Results

### Variation in microclimatic variables

**Figure 2:**
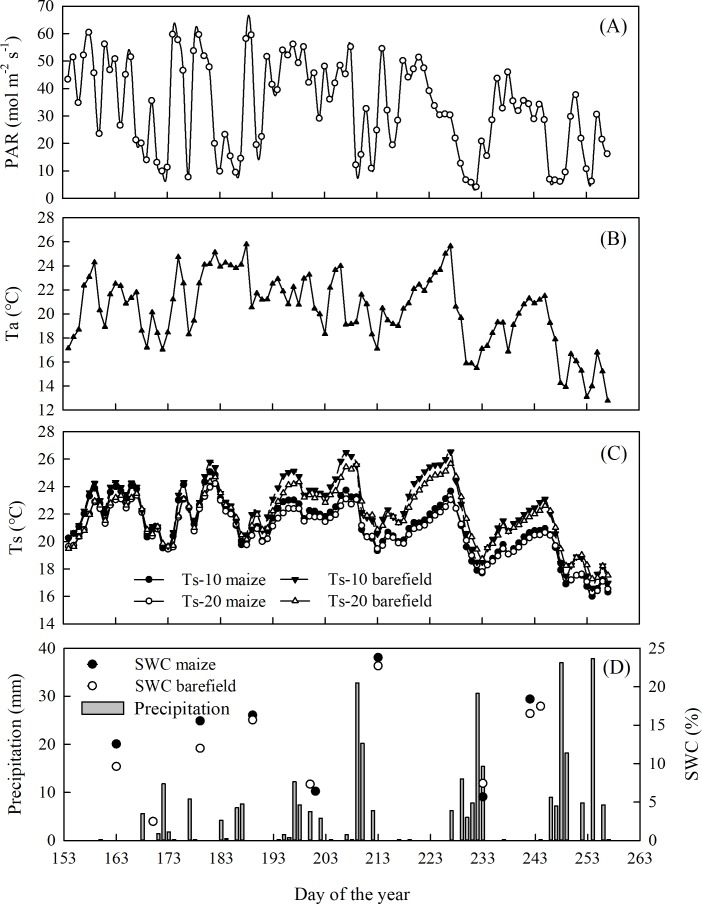
Seasonal variation of the environmental factors. (A) Photosynthetically active radiation (PAR), (B) air temperature, (C) soil temperature at 10 cm and 20 cm depth, (D) precipitation and soil water content (SWC) at 10 cm depth in the maize and bare.

The environmental conditions varied widely during the measurement period (Fig. 2). The mean Ta was 20.3 ± 2.9 °C, while the mean Ts at 10 cm were 22.3 ± 2.3 and 21.1 ± 2.1 °C in the bare land and the maize field, respectively (*p* >0.05). The corresponding mean soil moisture levels in the bare land and the maize field at the 10 cm depth were 14.0 ±1.0% and 15.4 ± 1.5%, respectively (*p* > 0.05). The total precipitation during the measurement period was 388.4 mm.

### Diurnal variation characteristics of the CO_2_ fluxes

**Figure 3:**
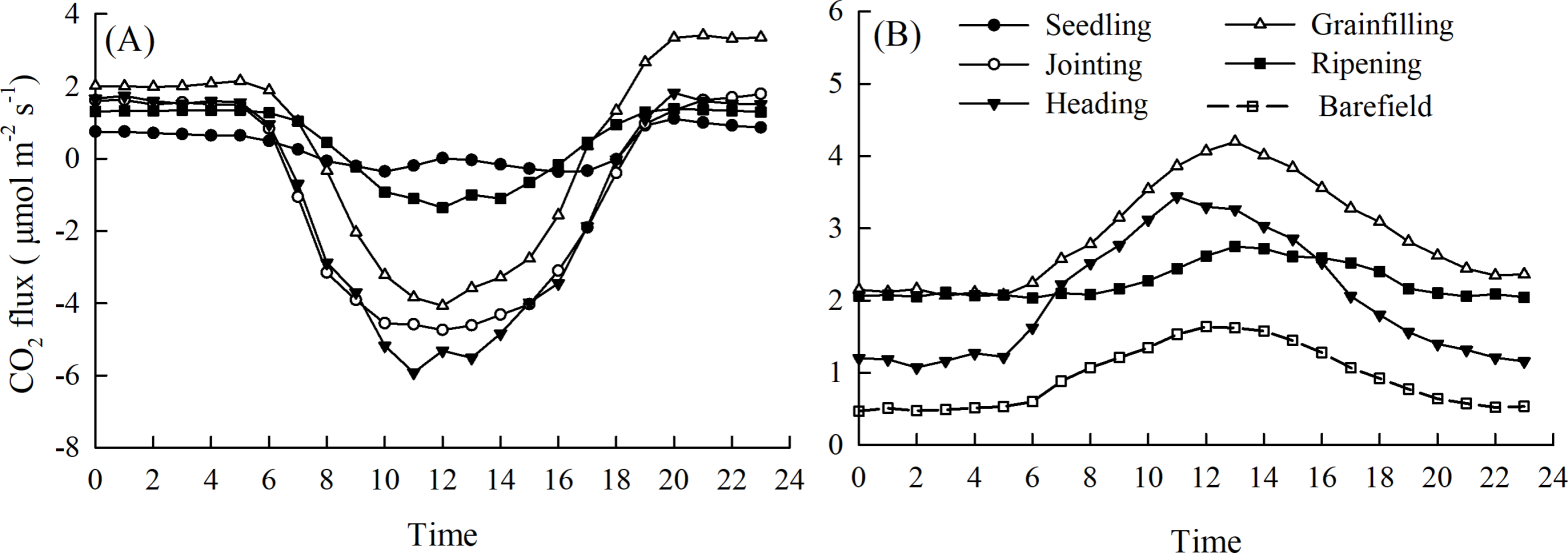
Diurnal variations of the CO _2_ flux during different growth periods of maize: (A) NEE-maize, (B) SR-maize and SR-barefield.

There were noticeable diurnal variations in the NEE in the spring maize ecosystem (NEE-maize) during each growth period, with positive values in the nighttime (19:00–6:00) and negative values during the day (7:00–18:00) (Fig. 3A). This was consistent with the trend in the PAR, and a short “noon break” occurred when the PAR intensity was at its maximum. The minimum daily NEE-maize values in the seedling, jointing, heading, grain filling, and ripening stages were –0.37, –4.74, –5.92, –4.07, and –1.36 μmol CO_2_ m^−^^2^ s^−^^1^, respectively. The diurnal variation in soil respiration (SR-maize) rates were best described with peak values in the heading, grain filling, and ripening stages of 3.44, 4.20, and 2.75 μmol CO_2_ m^−^^2^ s^−^^1^, respectively. The peak value of soil respiration rates in the bare field (SR-bare field) was 1.66 μmol CO_2_ m^−^^2^ s^−^^1^, which was lower than that for the SR-maize (Fig. 3B).

### Seasonal trend in CO_2_ fluxes during the maize growth period

#### Overall trend

**Figure 4:**
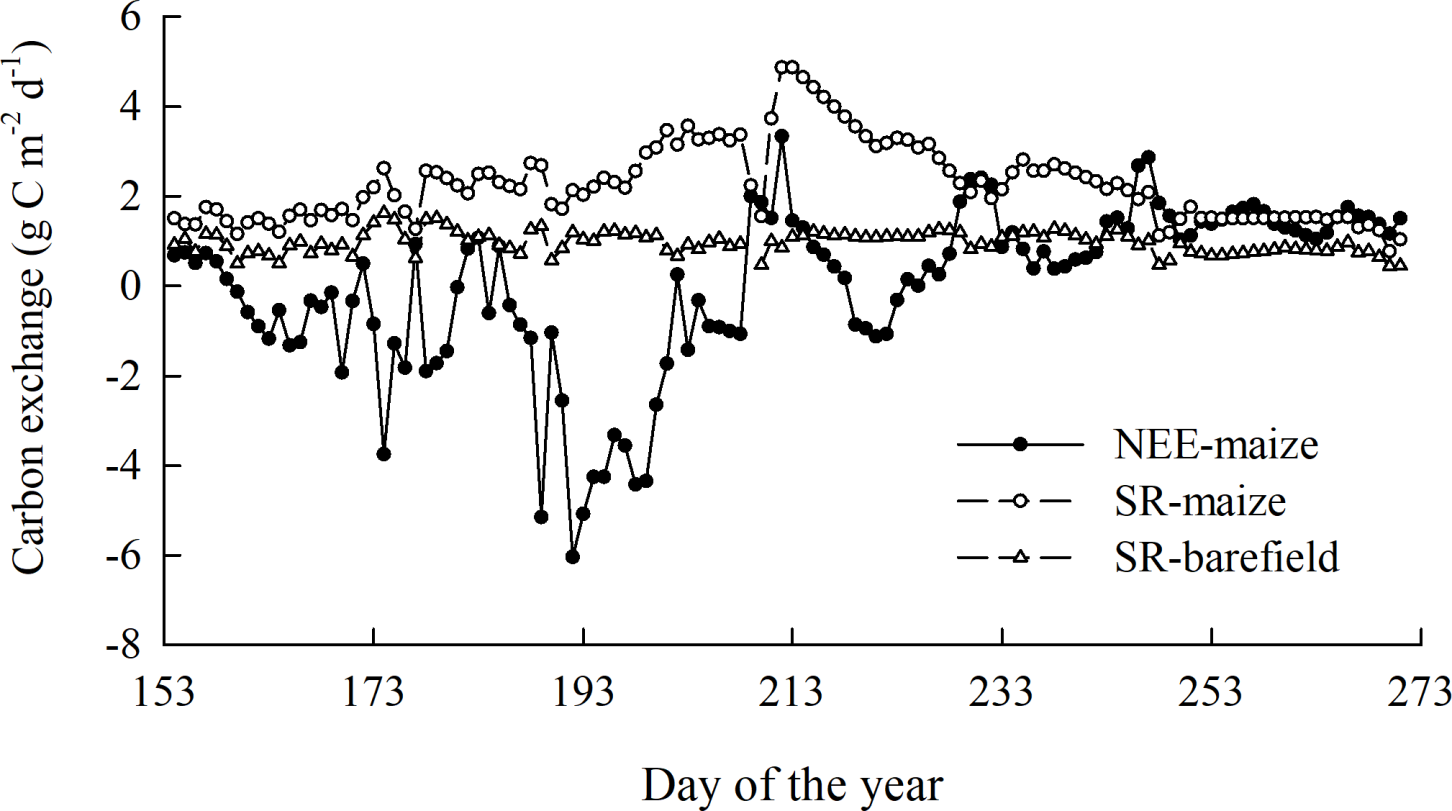
The overall trend of the CO_2_ flux during the observation period.

Throughout the observation period, the daily NEE-maize ranged from –6.04 to 3.33 g C m^−^^2^ d ^−^^1^, while the daily SR-maize and SR-bare field had values of 0.77–4.86 g C m^−^^2^ d^−^^1^ and 0.41–1.63 g C m^−^^2^ d^−^^1^, respectively. The cumulative NEE-maize (i.e., carbon uptake) and SR-maize (i.e., carbon emission) were –68.61 and 245.69 g C m^−^^2^, respectively, with corresponding soil carbon emissions in the bare field of 114.08 g C m^−^^2^. These results indicated a larger amplitude in the flux of the SR-maize than the SR-bare field, and the spring maize ecosystem functioned as a weak carbon “sink” throughout the growing season (Fig. 4). The carbon in the grain (C_*gr*_) was calculated as 234.60 g C m ^−^^2^. This figure considered the carbon released by the grains, and hence the maize field turned into a slight carbon source.

#### Carbon flux characteristics during different growth stages

**Figure 5:**
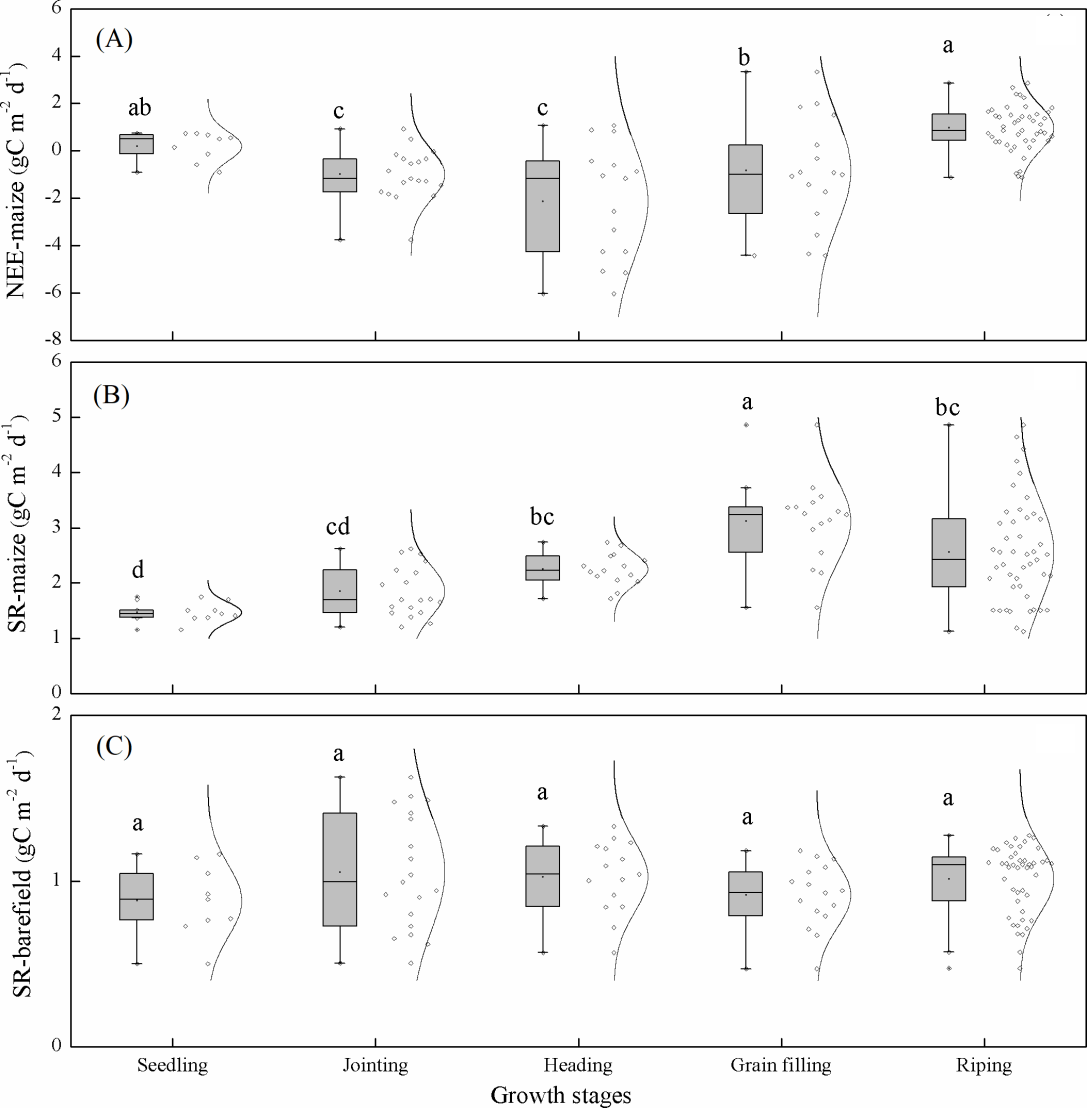
Daily average carbon exchange during each growth period: (A) NEE-maize, (B) SR-maize, and (C) SR-bare field. The dots and lines beside the box represent the carbon exchanges and their normal distribution. The error bars indicate the significance of differences among the different samples. The lowercase letters indicate the significance of differences among the growth stages (*p* < 0.05).

The cumulative NEE-maize values in the seedling, jointing, heading, grain filling, and ripening stages were 2.63, –19.85, –32, –16.76, and 65.26 g C m^−^^2^, respectively. To enable a more intuitive comparison, the averaged carbon fluxes during each growth stage were also calculated. The NEE-maize and SR-maize showed significant variations among stages. The NEE-maize during the heading stage was −2.13 ±  0.25 g C m^−^^2^ d^−^^1^ (Fig. 5A), and the SR-maize during the grain filling stage was 3.12 ± 0.70 g C m^−^^2^ d^−^^1^ (Fig. 5B). Therefore, they were significantly smaller and larger than those during other stages (*p* < 0.05). However, the SR-bare field showed no significant variations according to the above stages (Fig. 5C).

### The temperature sensitivity of NEE and soil respiration

**Figure 6:**
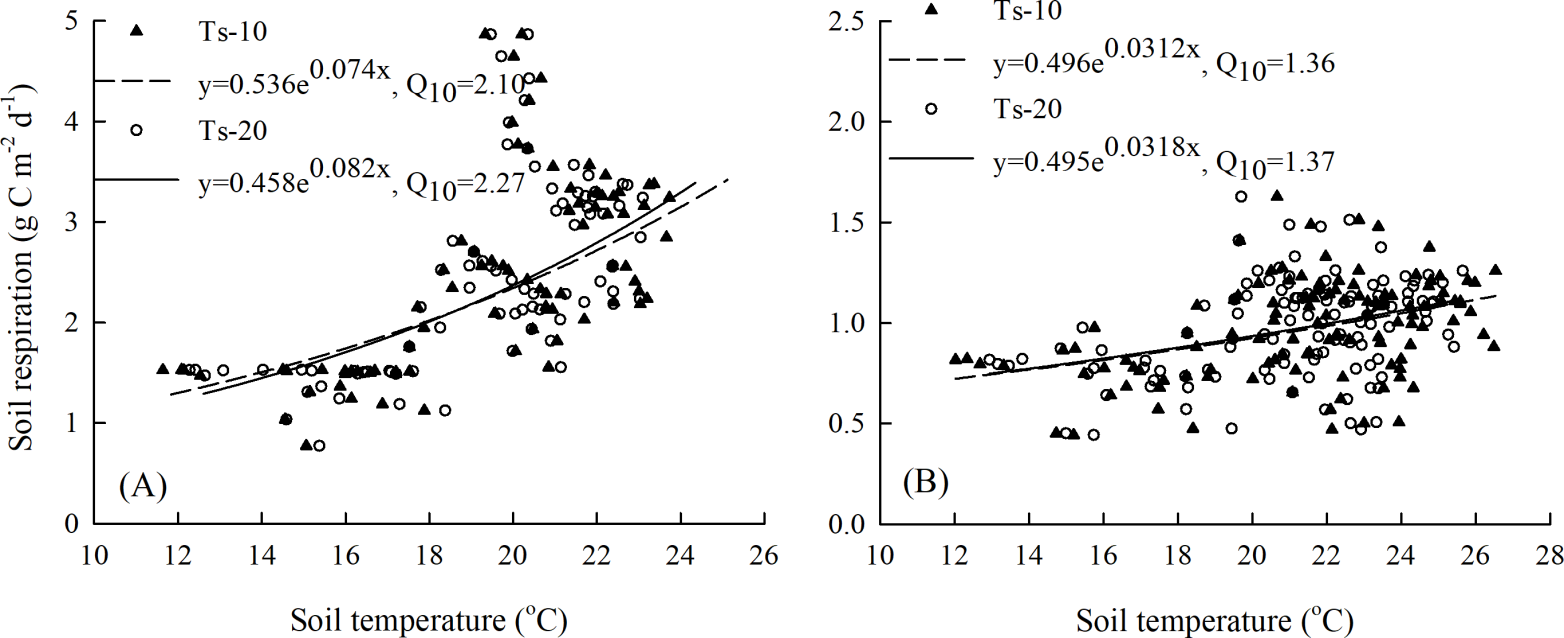
Relationships between SR and soil temperature at 10 cm and 20 cm depths: (A) maize field, and (B) bare field.

As Fig. 6 shows, the SR-maize values had significant positive correlations with soil temperature, for which the Q_10_ values were 2.10 and 2.27 at the 10 cm and 20 cm depths, respectively (Fig. 6A). The corresponding Q_10_ values were 1.36 and 1.37, respectively, for the SR-bare field (Fig. 6B). This suggested that the SR-maize was more sensitive to temperature than the SR-bare field, and also more sensitive to changes in soil temperature at the 20 cm depth.

**Figure 7:**
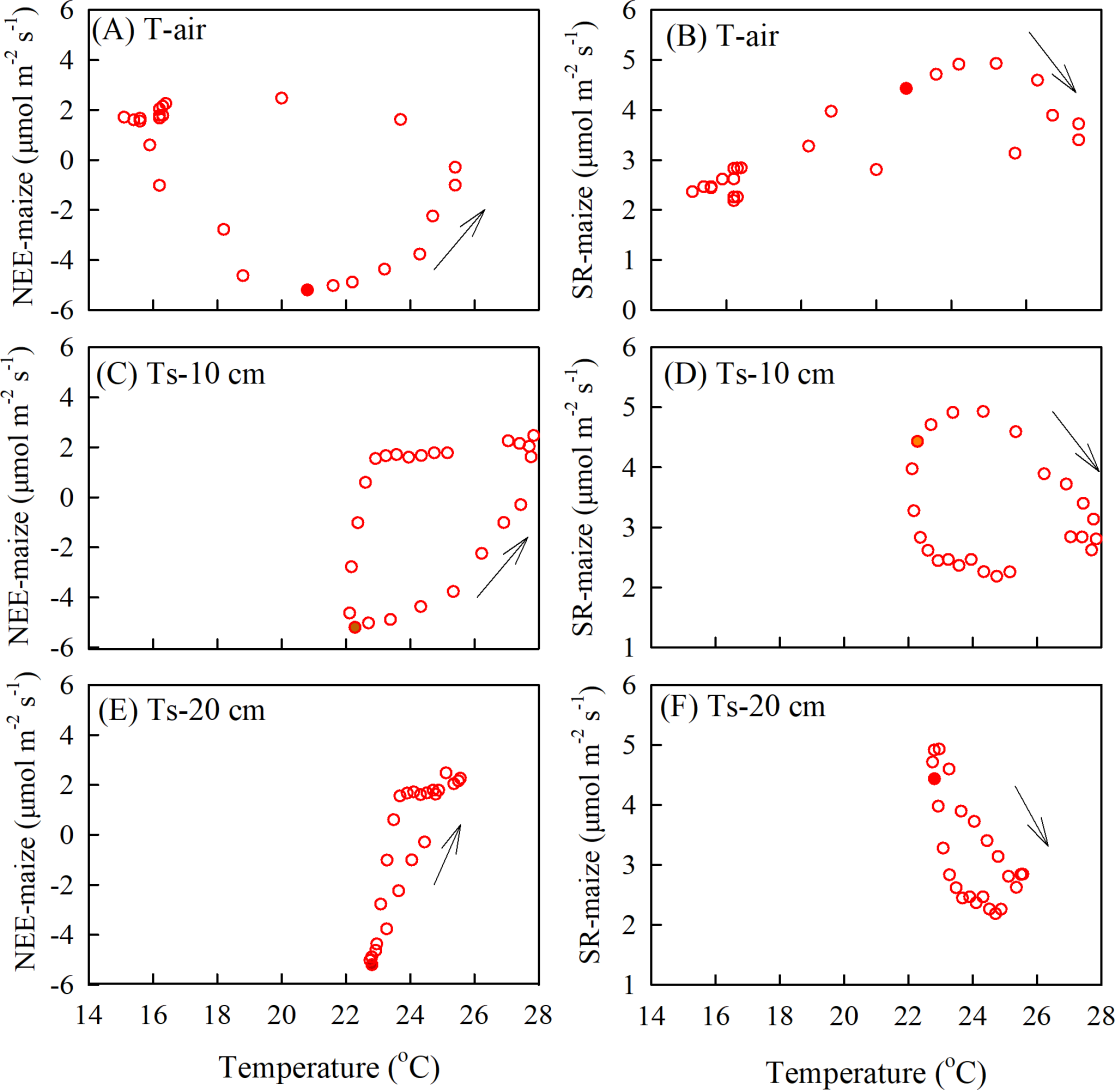
The diel hysteresis between the CO_2_ fluxes and temperature. ****(A), (C), and (E) indicate the NEE-maize with air temperature, and soil temperature at 10 cm, 20 cm depths; (B), (D), and (F) indicate SR-maize with air temperature and soil temperature at 10 cm and 20 cm depths. The solid points show time at 12 hours, and the arrows indicate the direction of hysteresis over time.

The relationship between soil respiration and soil temperature varied with the measurement depth, which was caused by an attenuation and phase shift in the soil temperatures with depth (Fig. 7). As the air temperature reached a daily maximum before the SR-maize and SR-bare field, the hysteresis loops rotated clockwise (Figs. 7A, 7B). However, when the soil temperatures peaked after the SR-maize and SR-bare field, the loops rotated counterclockwise (Figs. 7C–7F). This indicated that although the soil respiration was affected by temperature, the trend was not consistent with the single-peak trend of temperature, thereby resulting in the hysteresis.

### Response of CO_2_ flux to meteorological variables

**Figure 8:**
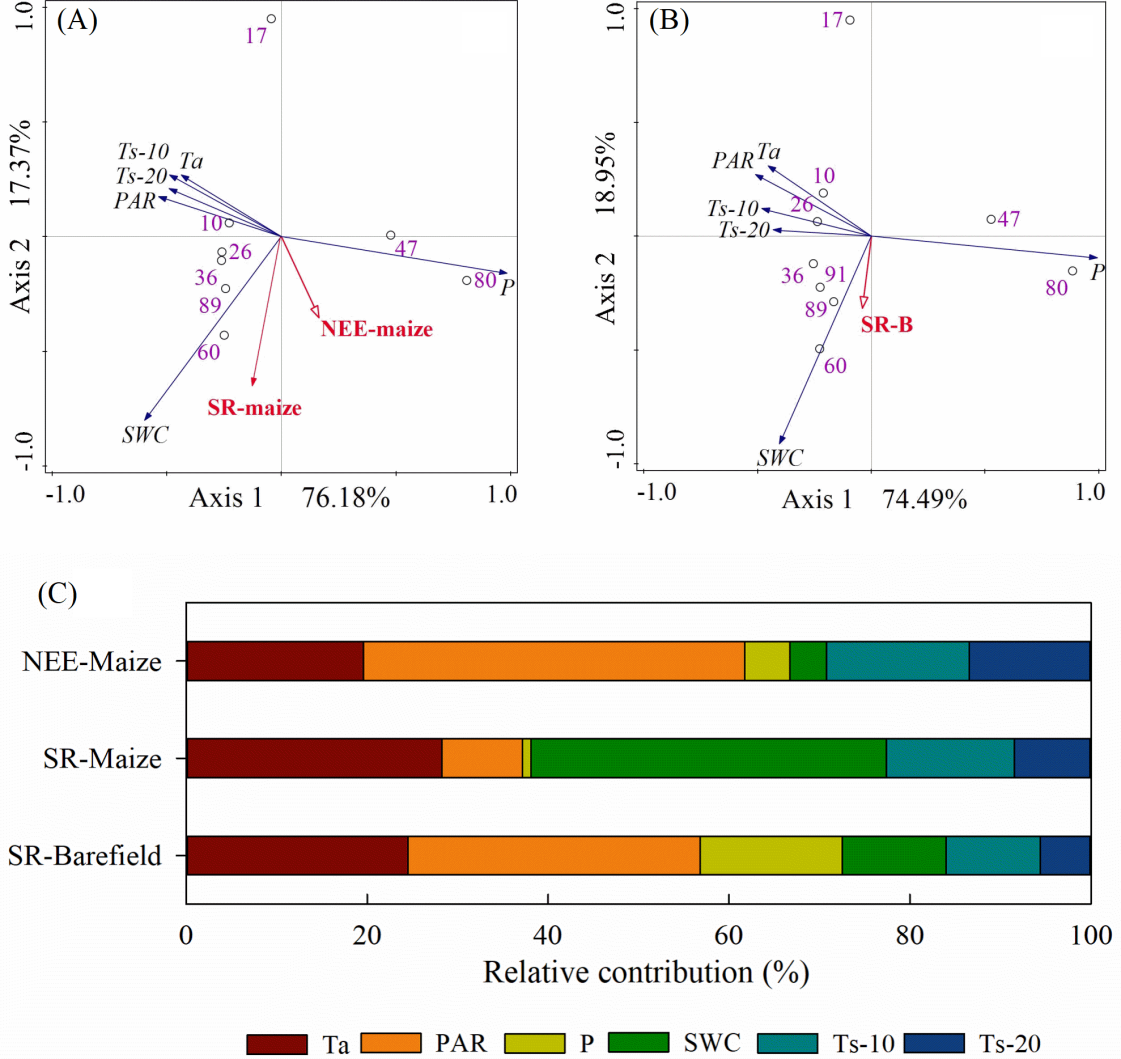
PCA analysis and the relative contribution of environmental variables to the CO_2_ flux: (A) PCA analysis on factors and NEE-maize, SR-maize, (B) PCA analysis on factors and SR-bare field, and (C) the relative contribution of the factors. Ta, air temperature; PAR, photosynthetically active radiation; P, precipitation; SWC, soil water content; Ts-10, soil temperature at 10 cm depth; Ts-20, soil temperature at 20 cm depth.

The correlations and PCA analyses showed that the NEE-maize had significant negative correlations with Ta, PAR, and Ts (all *p*-values < 0.01) but had positive correlations with P and SWC (*p*-values < 0.05) (Table 1, Fig. 8A). Conversely, both the SR-maize and SR-bare field were positively correlated with Ta, PAR, and Ts, yet negatively correlated with P. All of the correlations were significant at the 0.05 or 0.01 levels. However, for both SRs, the correlations with SWC were not significant (*p*-values** > 0.05) (Table 1, Fig. 8B).

The relative contribution of each potential influencing factor to the CO_2_ fluxes were determined, in which it was presumed that the contribution of all variables totaled 100% (Fig. 8C). The Ta and PAR jointly contributed 61.7%, 37.2%, and 56.8% to the NEE-maize, SR-maize, and SR-bare-field, respectively, and thus were considered the primary factors that affected the CO_2_ fluxes in the study area. The relative contribution of SWC varied widely among the different CO_2_ fluxes, and this result may have been related to the measurement frequency. Finally, the contributions of the Ts-20 were less than those of the Ts-10, which indicated that the influence of soil temperature decreased with increasing depth.

## Discussion

### Comparison between the automated chamber and other systems

Accurate measurements of CO_2_ fluxes are necessary for understanding carbon cycling in terrestrial ecosystems (Balogh et al., 2007). Although most carbon cycling studies are based on the eddy covariance technique, the chamber technique for gas exchange measurements (Reicosky & Peters, 1977) has some advantages. First, the source of the flux is clear, which is not possible in the eddy covariance technique, although the total flux can be calculated using a footprint analysis. Second, the observation area is relatively small, and it is easy to distinguish different land use types, thereby providing spatial physiological heterogeneity information for different vegetation types (Borchard et al., 2015). Finally, the lower cost acquisition compared to the eddy covariance technique is a significant factor in its use. In this study, observation plots for a maize field and bare field were both 10 m ×10 m, which were relatively flat, evenly tilled, and had little spatial variability or heterogeneity. Therefore, three chambers in each plot were representative of the CO_2_flux measurements.

To verify the accuracy of the automatic chamber technique, the method can be compared with other systems such as the portable chamber method (Li-8100) and the eddy covariance technique (Lavigne et al., 1997; Wohlfahrt et al., 2005; Myklebust, Hipps & Ryel, 2008; Wang et al., 2013a). In this study, the SR rate in the maize field ranged from 1.07 to 4.20 μmol CO_2_ m^−^^2^s^−^^1^ during the maize growth period. Wang et al. (2016) reported SR rates between 1.37 and 2.71 μmol CO_2_ m^−^^2^s^−^^1^, while Liang et al. (2018) reported values from 1.35 to 5.59 μmol CO_2_ m^−^^2^s^−^^1^ in maize fields in similar regions using a portable chamber (Li-8100) (Table 2). Similar values of the SR rate have been also reported using the same system. For instance, the values ranged from 2.10 to 2.46 μmol CO_2_ m^−^^2^s^−^^1^ for wheat in the same region, as reported by Zhang et al. (2011), and from 3.30 to 3.80 μmol CO_2_ m^−^^2^s^−^^1^ for a pine forest floor in the USA (Speckman et al., 2015). The NEE-maize ranged from −6.04 to 3.03 g Cm^−^^2^d^−^^1^ among different growth stages, which was in agreement with data reported by Lei & Yang (2010) in the North China Plain and Posse et al. (2014) in Argentina, who used the eddy covariance technique (Table 2). The consistency of the results of this study with previous studies indicates the reliability of this automated chamber system.

**Table 1:** The correlations between carbon fluxes and environmental factors.

Flux variables	Ta	PAR	P	SWC	Ts-10	Ts-20
NEE-maize	−0.431^**^	−0.632^**^	0.314	0.261^*^	−0.432^**^	−0.385^**^
SR-maize	0.277^**^	0.218^*^	−0.243^**^	0.379	0.193^*^	0.113^*^
SR-bare-field	0.419^**^	0.481^**^	−0.337^**^	0.122	0.273^**^	0.199^*^

**Notes.**

Ta air temperature PAR photosynthetically active radiation P precipitation SWC soil water content Ts-10 soil temperature at 10 cm depth Ts-20 soil temperature at 20 cm depth

* significant effect at *p* < 0.05.

** significant effect at *p* < 0.01, *N* = 104.

**Table 2:** Comparison of ranges in CO2 fluxes observed using different methods.

**Site**	**Method**	**Vegetation**	**SR rates** (µmol CO_2_ m^−2^ s^−1^)	**NEE** (g C m^−2^ d^−1^)	**Year**	**Source**
Shaanxi, China	Automatic Chamber (Li-820)	Maize	1.07∼4.20	−6.04∼3.33	2011	This study
Shaanxi, China	Automatic Chamber (Li-820)	Winter wheat	2.10∼2.46		2006–2007	Zhang et al. (2011)
Laramie, USA	Portable chamber (Li-820)	Pine forest	3.30∼3.80		2005–2011	Speckman et al. (2015)
Shaanxi, China	Portable chamber (Li-8100)	Maize	1.37∼2.71		2014–2015	Wang et al. (2016)
Hebei, China	Portable chamber (Li-8100)	Maize	1.35–5.59		2010	Liang et al. (2018)
Shandong, China	Eddy covariance (Li-7500)	Maize		−13.90∼3.65	2005–2009	Lei & Yang (2010)
Buenos Aires, Argentina	Eddy covariance (Li-7500)	Maize		−8.90∼4.00	2011–2012	Posse et al. (2014)
Shanxi, China	Eddy covariance (Li-7500)	Maize		−15.98 (peak value)	2014	Gao et al. (2017)
Texas, USA	Eddy covariance (Li-7500)	Maize		−14.78 (peak value)	2016	Wagle et al. (2017)

**Notes.**

SR rates soil respiration rates NEE the net ecosystem exchange of carbon; with the model of the infrared gas analyzer shown in bracket of each method

The peak values of the NEE-maize in the present study showed larger differences than the values reported by Gao et al. (2017) and Wagle et al. (2018), who used eddy covariance techniques (Table 2). Moreover, the cumulative NEE-maize and SR-maize in this study were –68.61 and 245.69 g C m^−^^2^, respectively, which was lower than those obtained by Li et al. (2006), Wang et al. (2013a) in the North China Plain and those obtained by Gao et al. (2017) in the Loess Plateau. Differences in elevation, precipitation, vegetation, period of time, observation scale, and the method of carbon flux determination are all possible causes of the above-mentioned differences. Therefore, further studies to compare the CO_2_ fluxes measured using an automated chamber and the eddy covariance technique are necessary to highlight the potential sources of error and enhance the reliability of the chamber system.

### CO_2_ fluxes in different land-use types

The automated chamber system in this work provided accurate and useful observations of CO_2_ flux at a plot scale and can therefore be used for investigations of other land-use types, such as in alpine meadows (Chen et al., 2014), forests (Liang et al., 2004; Takagi et al., 2009), and shrublands (Burrows et al., 2005). The daily SR-maize in this study was significantly larger than that of the SR-bare field during each maize growth stage (Fig. 5). This result indicated that land-use types had impacts on CO_2_ flux, which agreed with the findings of previous studies (Iqbal et al., 2008; Arevalo et al., 2010; Wang et al., 2016). Iqbal et al. (2008) reported significant differences in CO_2_ fluxes among four different land use types of subtropical red soil-paddies, orchards, woodlands, and uplands. Liu et al. (2016) also indicated that land use type had a significant effect on the diurnal variation in soil respiration, and naturally regenerated vegetation is the optimal vegetation type for reducing global warming. Different land-use types have various vegetation coverages, root biomasses, and soil physicochemical and microbial properties, which are all sources of differences in CO_2_ fluxes (Gu et al., 2018). However, in this study, only the soil respiration in the maize field and bare field were measured. Further research is required to cover more vegetation types, as well as their root and soil microbial information.

In terms of the regional carbon budget, the primary difference between agroecosystems and natural ecosystems is that crop grains are harvested and eventually consumed. Hence, they are finally transformed to CO_2_ and released back into the atmosphere (Li et al., 2006; Lei & Yang, 2010; Wang et al., 2013b). Thus, measuring the carbon removed during harvest coupled with the NEE is necessary for ecosystem carbon balance. This data can provide information on the total rate of organic carbon accumulation (or loss) from ecosystems (Chapin et al., 2006). The site studied in this work was a weak carbon source if grain carbon loss was considered during the maize growth period in 2011, which was similar to other reported results (Verma et al., 2005; Lei & Yang, 2010; Gao et al., 2017; Poyda et al., 2019). However, due to the limited time of observation, the accumulated data only revealed the carbon balance during the growing period. Follow-up observations should consider not only the CO_2_ flux during the growing period as well as the amount of biomass that is harvested, but also the presumable respired amount of CO_2_ during the fallow months throughout the year.

### Dependence on meteorological variables

Since NEE is the result of gross ecosystem carbon uptake in combination with ecosystem respiration, plant photosynthetic capacity and respiration intensity should have important impacts on NEE (Reichstein et al., 2005). When photosynthesis increased, the net CO_2_ flux was directed downward, whereas when the R_eco_ was high, and the net CO_2_ flux was directed upward (Fig. 3). Similar diurnal patterns have been described in previous studies (Hoffmann et al., 2015; Ohkubo, Nagata & Hirota, 2015). The relative contributions of Ta and PAR amounted to 61.7%, 37.2%, and 56.8% in the NEE-maize, SR-maize, and SR-bare field, respectively (Fig. 8). This result revealed the importance of these two factors for driving CO_2_ flux. Moreover, the pattern and amplitude of the diurnal trends of NEE and soil respiration changes with the seasons and depends on other environmental parameters, such as the leaf area index, in addition to the incident PAR (Chen et al., 2014).

Soil temperature, soil moisture, and their interaction largely control the temporal and spatial variations in soil respiration (Song et al., 2018). In a study of three European forests, Borken et al. (2002) found that soil temperature at a depth of 10 cm explained 73% to 86% of temporal variation in soil respiration. However, the influence of soil temperature varied in very wet or dry conditions. Chang et al. (2014) indicated that soil moisture was positively correlated with soil temperature when soil moisture exceeded the threshold 17%. In this study, correlations among soil respiration and soil temperature or soil moisture in the spring maize ecosystem and bare field were different, and the contributions of soil temperature to soil respiration were relatively small. Zhang et al. (2011) also reached a similar conclusion. This result may be explained by the inconsistent change in rainfall that affected soil moisture levels in our study area. The wetting and drying of soil can substantially influence CO_2_ emissions (Muhr et al., 2008). Furthermore, the positive or negative effect of each of these factors may not be individually explained because these factors are often strongly inter-correlated and co-vary with the soil organic matter content and root respiration, which are major sources of soil respiration (Song et al., 2018). In this study, the number of soil moisture measurements (seven times) during the maize growing season was limited, and the calculation results of the effect of water on CO_2_ flux did not reach a significant level. Therefore, the differences and driving mechanisms of the carbon balance process under the maize ecosystem and bare field require longer observations and more in-depth analysis.

## Conclusions

Both NEE and SR rate in maize ecosystems undergo a clear diurnal variation. CO_2_ flux is consistent with the trend of photosynthetically active radiation, and a short “noon break” occurs when the photosynthetically active radiation intensity is at its maximum. With the growth and development of maize plants, the carbon fluxes among their growth stages showed significant differences (*p* < 0.05), for which average values during the growth period were in the order of heading > grain filling > ripening > seedling-jointing stage. During the maize growth period in 2011, the overall NEE was −68.61 g Cm^−^^2^, which represented as a weak carbon sink. However, it changed to a small carbon source if grain harvest was considered. Ta and PAR were the primary factors that affected CO_2_ flux in the cornfield, as evidenced by the current study results that showed Ta and PAR contributed 61.7%, 37.2%, and 56.8% to the NEE-maize, SR-maize, and SR-bare-field, respectively. Therefore, improving the photosynthetic efficiency of spring maize in this region would be helpful to improve the carbon sink function of the crop ecosystem, which is of great significance for the regional carbon cycle.

##  Supplemental Information

10.7717/peerj.8994/supp-1 Supplemental Information 1 Raw data

## Additional Information and Declarations

### Data Availability

The following information was supplied regarding data availability: The raw measurements are available in the Supplemental File.
